# The Carotid Sinus Nerve and the First English Translation of Hering's Original Research on this Nerve

**DOI:** 10.7759/cureus.3898

**Published:** 2019-01-16

**Authors:** Mohammadali M Shoja, Rabjot Rai, Stefan Lachkar, Seleipiri Iboroma Akobo, Emre Yilmaz, Marios Loukas, Emanuela Binello, Mehrnoush Gorjaian, Christoph J Griessenauer, Joe Iwanaga, R. Shane Tubbs

**Affiliations:** 1 Surgery, University of Texas Medical Branch, Galveston, USA; 2 Anatomy, St. George's University School of Medicine, St. George's, GRD; 3 Anatomy, Seattle Science Foundation, Seattle, USA; 4 Miscellaneous, Tabriz University of Medical Sciences, Tabriz, IRN; 5 Surgery, Swedish Neuroscience Institute, Seattle, USA; 6 Medical Education and Simulation, St. George's University School of Medicine, St. George, GRD; 7 Neurosurgery, Boston University School of Medicine, Boston, USA; 8 Neurosurgery, Barrow Neurological Institute, Phoenix, USA; 9 Neurosurgery, Geisinger Medical Center, Danville, USA; 10 Medical Education and Simulation, Seattle Science Foundation, Seattle, USA; 11 Neurosurgery, Seattle Science Foundation, Seattle, USA

**Keywords:** vagus nerve, baroreceptors, glossopharyngeal nerve, carotid sinus nerve, historical

## Abstract

This paper provides a brief depiction of the life and achievements of the most iconic experiments of Heinrich Ewald Hering. The authors herein have presented a translation of his paper on the carotid sinus nerve in English; the original paper by Heinrich Ewald Hering, titled “Ueber die Wand des Sinus caroticus als Reizempfänger und den Sinusnerv als zentripetale Bahn für die Sinusreflexe” (1924), provides a detailed account of his experimental process and findings. He recognized that the sinus reflexes are mediated by a branch of the glossopharyngeal nerve (CN IX).

## Introduction

Understanding the medical history behind medical eponyms allows clinicians and scientists to appreciate the pathway to such discoveries. Medical eponyms allow for acknowledgment of the contributions made by those in various fields—this includes Heinrich Ewald Hering (1866-1948) whose work coined the term "Hering’s nerve or sinus nerve of Hering". Hering's nerve is a branch of the glossopharyngeal nerve (CN IX) that innervates the baroreceptors of the carotid sinus and the chemoreceptors in the carotid body. Herein, we have discussed his bibliography and translated his paper on the carotid sinus nerve into English.

## Materials and methods

The literature search was performed using the PubMed database with a focus on articles including descriptions of the Hering's nerve and the sinus nerve. Heinrich Ewald Hering's classic paper on the carotid sinus nerve was originally written in German and was translated into English by the authors. 

## Results

Heinrich Ewald Hering (1866-1948)

**Figure 1:**
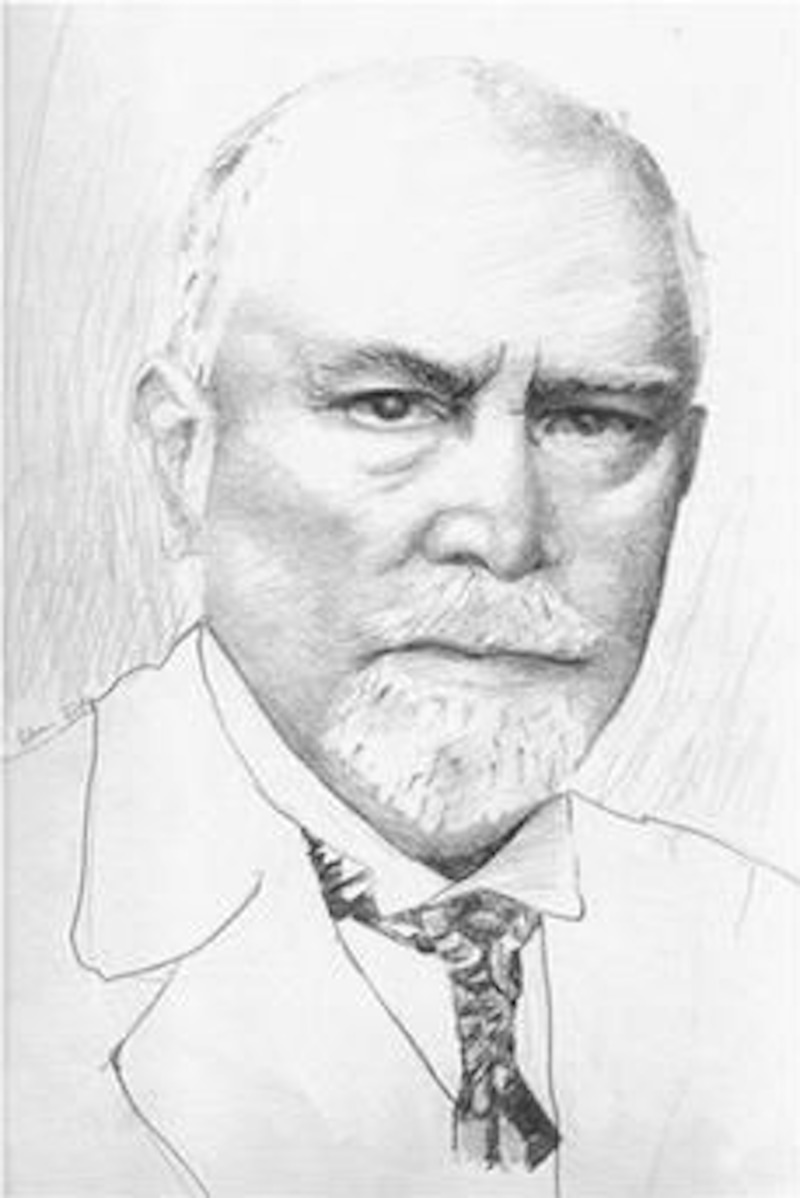
A sketch of Heinrich Ewald Hering by Salva M. Shoja

Heinrich Ewald Hering (Figure 1) was born on May 3, 1866 in Vienna to renowned physiologist Ewald Hering, the rector of the German University in Prague [1-2]. The younger Hering became interested in cardiovascular physiology as a result of Czermak’s self-experiment on the negative chronotropic effects of the vagus nerve on the heart published in 1866 [2].

Hering matriculated at the University of Prague where he initially studied the innervation of skeletal muscles that later stimulated his interest in the physiology and pathology of the heart and great vessels, specifically their correlation with regard to pulse rate and activity of the chambers of the heart [3]. In 1895, while in Czechoslovakia, Hering reported an increase in cardiac output due to increases in the heart rate. This response occurred secondary to the withdrawal of vagal tone during the initiation of exercise [1,3].

Hering went on to become a professor of general and experimental pathology at the University of Prague. Later, he accepted an invitation to become the Director of the Institute of Pathologic Physiology at the University of Cologne in 1913 [2].

**Figure 2:**
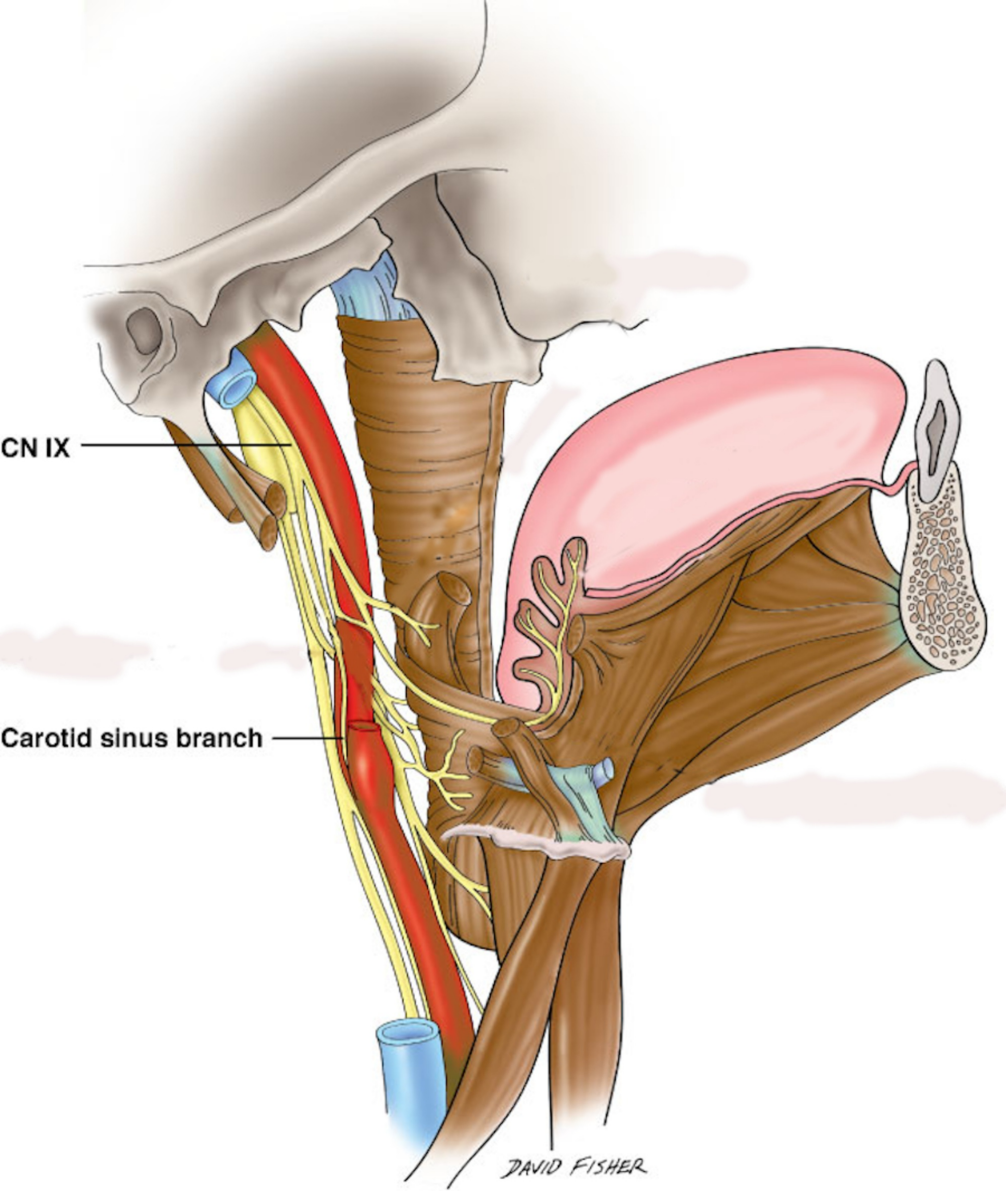
A schematic drawing of the carotid sinus nerve

Hering’s passion for cardiovascular function can be seen in his work on the carotid sinus reflex, independent action of the auricles and ventricles, and the analysis of the pulsus irregularis perpetus—currently known as atrial fibrillation, a phenomenon he studied using the polygraph while at the German University in Prague in 1903 [2-3]. Hering continued his studies on atrial fibrillation and other arrhythmias with the emergence of the Edelman electrocardiograph in 1907 [1-3]. Hering's work on the carotid sinus (Figure 2) and the receptors that dwell therein were summarized and the data put in a monograph containing results of numerous other experiments he had worked on—both on animals and humans—which he began publishing on February 26, 1924 [2]. As a result of his research and academic contributions, he became the first president of the German Society for Heart and Circulation Research [2].

Later, Hering expanded on the baroreceptor reflex concept, which was initially proposed and worked on by Elias Cyon and Carl Ludwig. They proposed that the baroreceptors were located in the heart but propagated information to the brain, which in turn influenced the vascular tone via the vagus nerve. Their report was focused on the depressor nerve and its effect on cardiac overload and failure due to a causal ebbing in heart rate and reduction in total peripheral resistance and the resulting unloading of the left ventricle. They were bemused at the increase in heart rate from direct stimulation of the heart as opposed to the decreasing heart rate from the depressor nerve. Hering’s response to this report was one of criticism, mostly for the term "depressor nerve", which he thought did not fully capture the true essence of the functioning of the nerve, as the depressing effect they reported was only observed by artificially stimulating the nerve [1-5].

Hering was elected into the Leopoldina, the German National Academy of Sciences in 1932; he was given emeritus status in 1934 for his work on the pressure receptors; and, although he did not win, he was nominated for the Nobel Prize for medicine and physiology in 1937. In 1948, at the age of 83, Hering died in Mecklenburg, Germany [2].

## Discussion

English translation of Hering’s classic paper on the carotid sinus nerve

The translation below is from Hering’s 1924 paper, “Ueber die Wand des Sinus caroticus als Reizempfänger und den Sinusnerv als zentripetale Bahn für die Sinusreflexe,” which translates to “About the wall of the carotid sinus as a stimulus-receiver and the sinus nerve as a centripetal pathway for the sinus reflexes,” and is where Hering reported his findings regarding the sinus nerve.

Using the so-called Czermak’s vagal pressure test, I found that its effects are not the result of pressure on the vagal nerve and the mechanical irritation of its heart inhibitory fibers, but due to a reflex from the carotid sinus, which is located at the origin of the internal carotid artery. I also found a second reflex emanating from the carotid sinus, which has a quite vigorous effect on reducing the blood pressure. Both reflexes, the heart-inhibitory and vasodilatory, disappeared after denervation of the carotid sinus. Denervation was accomplished by dissection and removal of the tissues located within the carotid bifurcation. Furthermore, I established that the superior laryngeal nerve did not play a role in the sinus reflexes or with the carotid compression test. I mentioned in a note: “The nerves that were rendered nonfunctional with denervation of the sinus reflexes come from the superior cervical ganglion of the sympathetic nervous system. I made this observation in a larger dog.” Based on these pure anatomical findings, it cannot be clearly delineated whether these centripetal sinus nerves originate from the sympathetic ganglion or to the adjacent vagal nerve; thus, I decided to further investigate the course of the sinus nerve.

I dissected the superior cervical ganglion in rabbits, which is simpler than in dogs, and found that the sinus reflexes remain after removal of the superior cervical ganglion of the sympathetic nervous system.

It was very unlikely that such observation would be different in the dogs. But before I performed the same experiment on dogs, I remembered a remark from my former teacher Ph. Knoll in 1885 that the glossopharyngeal nerve was a depressor nerve. That information inspired me to expose the glossopharyngeal nerve immediately past its exit from the skull base in dogs. In the subsequent experiments in both dogs and rabbits, the division of a branch of the glossopharyngeal nerve resulted in the disappearance of the above-mentioned reflexes. It is the first branch of the glossopharyngeal nerve visualized past its exit from the skull base and I will refer to this nerve as the sinus nerve.

With transection of the sinus nerves, the carotid sinus is denervated, the blood pressure increases, and the clamp response is abolished. This means the heart rate and arterial blood pressure do not drop when the sinus clamp is applied. Similarly, the arterial blood pressure and heart rate no longer increase after occlusion of the common carotid artery.

Thus, the sinus nerve represents an analog for the depressor nerve, which in rabbits run isolated along the same course as the sinus nerve.

As the transection of the sinus nerve abolishes the sinus reflexes, irritation of the nerve can provoke these reflexes. Electrical stimulation of the central stump of the transected sinus nerve triggers the same response as the stimulation of the carotid sinus. The effects of stimulation of the sinus nerves are stronger than the stimulation of a depressor nerve. Historically, the description of depressor nerve is opposite from the description of the sinus nerves. The depressor nerve was first identified and, much later, the aortic root was recognized as its starting point. The sinus nerve was identified half a year after the origin of the reflexes, the carotid sinus, was found.

As far as I know, there is no defined term describing the sinus nerves despite its depiction in an anatomical textbook where it appeared in Figure 776 of Spalteholz’s handbook as a branch of the glossopharyngeal nerve, which stretches to the sinus region of the internal carotid artery and has a connection to the vagal ganglion. There are some additional observations that warrant mentioning.

With the injection of warm Ringer’s solution, normal saline, or distilled water into the periphery of the carotid bifurcation, the blood pressure drops depending on to the quantity of the fluid and the rate of injection. With each injection, the animal twitches. The blood pressure will drop even if adrenaline is injected since the quality of the fluid does not matter.

This explains the dual, temporally space vagal effect that Biedl and Reiner observed upon injection of adrenal extract into the periphery of the carotid bifurcation of the dog; the initial effect triggers the sinus reflexes that are abolished with denervation.

In some cases, I noticed increased excitability of the carotid sinus. A rabbit (experiment 128), in which fourteen days prior both common carotid arteries were ligated proximal to the thyroid artery and both of the depressor nerves had been transected six days afterward, showed increased sensitivity of the carotid sinus. During the clamp test, the blood pressure remained low for a long time, for example, 12 minutes, and increased again after reopening of the clamp, while otherwise during the presence of the clamp, the pressure rise occurs sooner. A pulling force at the carotid bifurcation can also keep the pressure low for some time.

In the previously mentioned rabbit (experiment 128), both vertebral arteries were very tortuous. Cross-clamping resulted in a significant increase in pressure, which gradually decreased in waves. The breathing pattern corresponded to the waves. Peak breathing occurred more frequently and at greater amplitude, while wave trough breathing was less frequent and of smaller amplitude.

I also noticed an increase in sensitivity after injection of 5% normal saline into the periphery of the carotid bifurcation. It appeared as if the pressure reduction during the injection was greater with consecutive injections at certain time intervals and on clamp test, the pressure reduced more significantly.

In one rabbit, I observed a mean blood pressure of 125 mm Hg 14 days after the denervation of both sinuses and depressor nerves, a markedly higher blood pressure than usual (usually around 90 mm Hg).

In rabbits, I could clearly discriminate between the effects of the larynx pressure test and the sinus pressure test by transection of both superior laryngeal nerves.

At this point, I would like to emphasize the mechanical sensitivity of the depressor nerve in rabbits. Even careful lifting of the transected nerves from the wound, the positioning on the electrodes, etc., all resulted in the reduction of the pressure. Even a subtle stretch of an uncut nerve often results in very strong effects.

## Conclusions

Heinrich Ewald Hering’s discovery of a nerve branch arising from the glossopharyngeal nerve commonly referred to as the carotid sinus nerve (Hering’s nerve) further articulated how stimulation of the carotid body and sinus arose. Stimulation of the carotid body and sinus via Hering’s nerve has pertinent clinical significance in response to regulation of blood pressure and hypoxia. The translation of Hering’s paper highlights his findings of the carotid sinus nerve and its role in the regulation of blood pressure, which further contributes to the field of cardiovascular medicine.
